# Mechanical Thrombectomy Using Double Stent Retriever Technique for Acute Ischemic Stroke Following Embolism From the Pulmonary Vein Stump After Left Upper Lobectomy: A Case Report

**DOI:** 10.7759/cureus.56610

**Published:** 2024-03-20

**Authors:** Haruki Hirata, Yuta Kaneshiro, Yumiko Urano, Keiji Murata

**Affiliations:** 1 Department of Neurosurgery, Shimada General Medical Center, Shimada, JPN

**Keywords:** acute ischemic stroke (ais), double stent retriever technique, mechanical thrombectomy, left upper lobectomy, pulmonary vein stump

## Abstract

Acute ischemic stroke (AIS) following pulmonary lobectomy, which is traditionally attributed to air embolism and atrial fibrillation (AF), may occur after thrombus formation in the pulmonary vein stump (PVS). Herein, we document the mechanical thrombectomy (MT) of a carotid bifurcation occlusion post-left upper lobectomy (LUL) to manage AIS.

A 76-year-old male with a history of diabetes, dyslipidemia, and a treated dural arteriovenous fistula at the transverse sigmoid junction, with no history of AF, successfully underwent LUL for a pulmonary tumor. The patient independently walked on postoperative day 1. He developed right hemiparesis and total aphasia on the morning of the second day after surgery, which was discovered by the nursing staff. A magnetic resonance imaging (MRI) confirmed an occlusion of the left common carotid artery (CCA). Tissue plasminogen activator (t-PA) was not administered owing to recent surgery. An urgent MT using multiple MT techniques carried out 90 minutes after the discovery of symptoms only achieved partial recanalization. Subsequently, a double stent retriever technique (DSRT) addressed the occlusion in the common and cervical internal carotid artery (ICA). Following this, a T occlusion was encountered, which was addressed with a combined approach using a single stent retriever (SR), achieving a thrombolysis in cerebral infarction (TICI) grade 2b result. However, postoperative aphasia and severe right hemiparesis remained. Postoperative imaging showed a significant left cerebral hemisphere infarction and a thrombus in the PVS. Oral edoxaban was administered, and PVS thrombosis did not recur. The patient was transferred to a rehabilitation facility 190 days post-embolization with a modified Rankin Scale score of 4.

In this report, we demonstrate the challenging case of the DSRT in addressing AIS after LUL, which led to the formation of a massive thrombus and occlusion of the carotid artery, as revealed by the PVS. This case emphasizes the importance of collaborative efforts between thoracic surgeons and all staff involved in stroke care in managing such complex scenarios.

## Introduction

Acute ischemic stroke (AIS) post-pulmonary lobectomy may arise from air embolism and atrial fibrillation (AF). Additionally, pulmonary lobectomy carries a potential risk of AIS due to embolization from the pulmonary vein stump (PVS), with postoperative stroke occurring in 1.57% of patients who undergo pulmonary lobectomy or pneumonectomy [1]. It is crucial to distinguish between the overall incidence of postoperative stroke and the detection of thrombosis in the PVS. Specifically, left upper lobectomy (LUL) not only results in a longer PVS compared to other lobectomy types but also has been associated with a 13.5%-17.9% incidence of thrombosis in the PVS on imaging. This is notably higher than the 3.3%-3.6% detected in patients undergoing any lobectomy [2,3], suggesting that LUL or left pneumonectomy significantly increases the risk of stroke to 5.17% [1]. These findings underscore the importance of recognizing the differentiated risks between clinical outcomes of stroke and imaging findings of PVS thrombosis [1-3]. While most research on this topic comes from thoracic surgeons, reports from professionals such as neurosurgeons and neuroradiologists are rare [4-6].

However, it is important for medical professionals other than thoracic surgeons to also share their experiences. This is because quick diagnosis and action to restore blood flow are critical after the problem starts. In addition, patients who have undergone pulmonary lobectomy cannot be treated with the usual stroke medicine, tissue plasminogen activator (t-PA), unlike typical AIS treatments.

There has also been an increase in reports regarding the double stent retriever technique (DSRT) for the treatment of challenging cases of AIS. This approach involves utilizing two stent retrievers (SRs) during thrombectomy to attempt the removal of clots [7,8]. Our report features a challenging instance where DSRT was employed to address an occlusion at the carotid bifurcation, which was caused by an embolism from the PVS after LUL. Despite a less-than-favorable clinical outcome, in our experience, DSRT was more useful than other MT techniques in removing the large thrombus.

## Case presentation

A 76-year-old male was referred to the thoracic surgery division of our hospital for diagnostic assessment of an upper left pulmonary tumor initially detected by chest radiography screening. He had well-controlled diabetes mellitus, dyslipidemia, and a dural arteriovenous fistula at the transverse sinus that had been effectively occluded via endovascular coil embolization. He had been smoking 30 cigarettes daily for more than 50 years until the age of 70 years. He had no prior episodes of AF or cardiac conditions. Computed tomography (CT) identified a consolidated mass in his left upper pulmonary lobe, indicative of neoplasm. He underwent an LUL via video-assisted thoracoscopic surgery (VATS). The surgery was completed as scheduled. AF was not detected during or after the procedure. Heparin administration was not conducted considering the potential for PVS thrombosis, instead opting for monitoring and regular observation. He had no neurological impairment and could walk from the first postoperative day until six hours prior to detecting the abnormality. A pathological diagnosis of solid adenocarcinoma (pT1bN0M0 pStageⅠA2 as defined by the seventh edition of the TNM classification for lung cancer) was made using biopsy findings.

**Figure 1:**
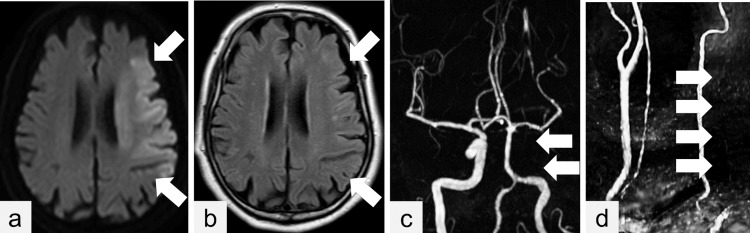
Head magnetic resonance imaging (a) Head magnetic resonance imaging shows increased signal intensity in the left middle cerebral artery territory on diffusion-weighted imaging (arrows). (b) This region does not exhibit increased signal intensity on fluid-attenuated inversion recovery imaging (arrows). (c and d) Magnetic resonance angiography shows an absence of flow in the left common carotid artery and internal carotid artery (arrows).

The second morning after the operation, he was found unconscious. His blood pressure and heart rate were within normal ranges, and his consciousness level on the Japan Coma Scale was 30. On examination, he had severe right hemiparesis, sensory disturbance involving the face, total aphasia, and a National Institute of Health Stroke Scale score of 22. CT of the head revealed a hyperdense area within the middle cerebral artery (MCA) with no evidence of early ischemic changes and air within the brain. Further examination via magnetic resonance imaging (MRI) showed increased signal intensity in the left MCA territory on diffusion-weighted imaging (DWI) that was not seen on fluid-attenuated inversion recovery (FLAIR) imaging (Figure 1a, 1b). Magnetic resonance angiography (MRA) did not detect any flow in the left common carotid artery (CCA) and internal carotid artery (ICA), but there was minor flow in the left A1 segment and MCA due to collateral circulation from the anterior communicating artery (Acom) (Figure 1c, 1d). A diagnosis of left CCA occlusion with DWI/FLAIR mismatch was made one hour after detecting the abnormality, and a neurosurgeon was consulted for the first time.

**Figure 2:**
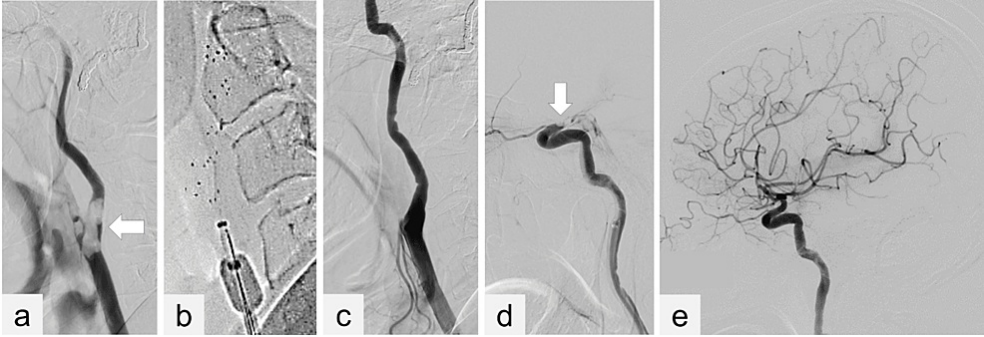
Digital subtraction angiography (a) DSA from the left CCA shows the large embolus at the carotid bifurcation (arrow). (b) The double SR technique. Two devices of Solitaire 6 × 40 mm SR are deployed on parallel across the embolus, and a REACT 71 AC is placed as near to the clot as possible. Both SRs and AC are pulled back together under continuous aspiration. (c) DSA from the CCA shows recanalization of the cervical ICA and (d) T occlusion at the top of the ICA (arrow). (e) The final DSA from the CCA shows partial recanalization of the ICA to the middle cerebral artery. DSA: digital subtraction angiography, CCA: common carotid artery, SR: stent retriever, AC: aspiration catheter, ICA: internal carotid artery

A tissue plasminogen activator (t-PA) was not administered owing to the patient's recent surgical history. Ninety minutes following the discovery, an urgent MT was performed under local anesthesia. First, a 9-French long sheath was inserted into the right femoral artery. A 9-French balloon guiding catheter (BGC) (Optimo; Tokai Medical Products, Aichi, Japan) was navigated to the CCA via the femoral approach. Digital subtraction angiography (DSA) from the left CCA revealed a large embolus at carotid bifurcation, extremely slow flow of cervical ICA, and no intracranial ICA (Figure 2a). Attempts made to directly remove the clot via aspiration through the BGC were unsuccessful. Next, Phenom 21 microcatheter (Medtronic, Minneapolis, MN, USA) was navigated through the blockage with a microguidewire (CHIKAI black 14 soft tip; Asahi Intec, Nagoya, Aichi) via the REACT 71 aspiration catheters (AC) (Medtronic, Minneapolis, MN, USA). Solitaire FR 6 × 40 mm SR (Medtronic, Irvine, CA, USA) was deployed, and after temporary blockage of CCA flow, the Solitaire was retrieved via aspiration using AC. Nonetheless, two attempts of the combined thrombectomy technique did not extract the thrombus. Consequently, we conducted the double SR technique in the next pass. Two devices of Solitaire 6 × 40 mm SR (Medtronic) were deployed parallel across the embolus, and AC was placed as near to the clot as possible (Figure 2b). Both SRs and AC were gradually pulled back together under continuous aspiration, and some hard clots were retrieved. The BGC had no flow reversal, so it was also removed, and we retrieved several clots from it. DSA from the CCA showed recanalization of the cervical ICA but T occlusion at the top of the ICA (Figure 2c, 2d). After two attempts at recanalization using a technique that combined a Solitaire 6 × 40 mm SR (Medtronic) and REACT 71 (Medtronic), partial recanalization of the ICA to the MCA was achieved (Figure 2e). The thrombolysis in cerebral infarction (TICI) grade was 2b. The total time from femoral access to recanalization was 100 minutes.

**Figure 3:**
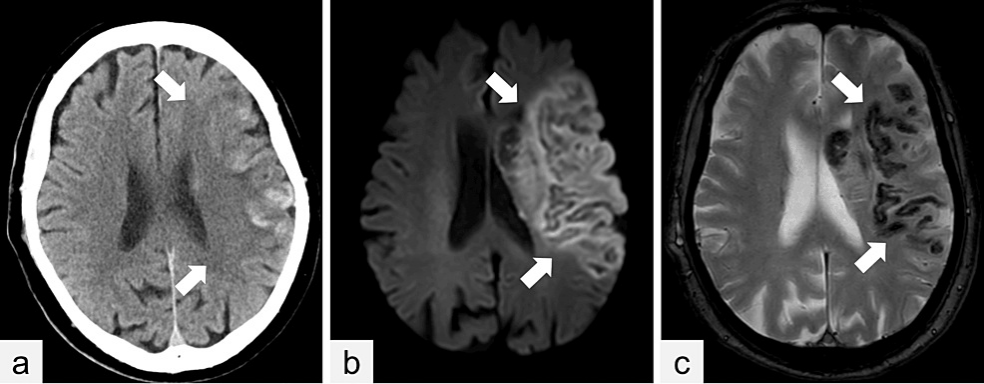
Postoperative computed tomography and magnetic resonance imaging (a) Postoperative computed tomography imaging shows a hypodense region within the left cerebral hemisphere (arrows). (b) Postoperative magnetic resonance imaging shows an extensive middle cerebral artery territory infarction on diffusion-weighted image (arrows) and (c) intracerebral hemorrhage on T2 image (arrows).

**Figure 4:**
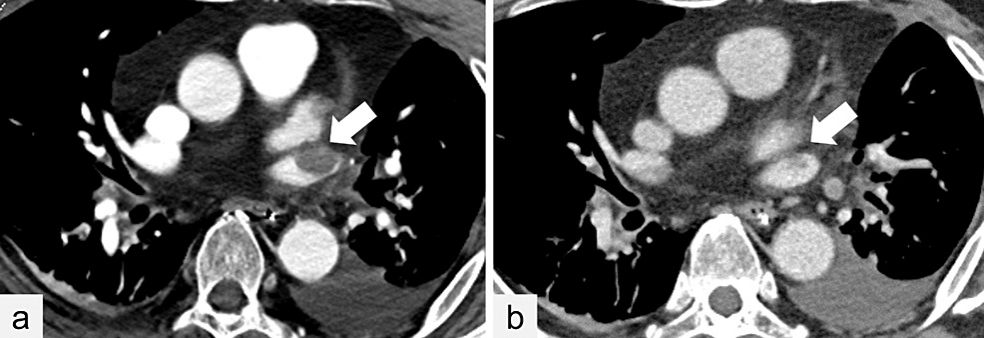
Contrast-enhanced computed imaging of the chest (a) Contrast-enhanced computed imaging eight days after the LUL shows a 20 mm thrombus in the pulmonary vein (arrow). (b) Eighteen days post-LUL, a reduction in thrombus dimension is detected (arrow). LUL: left upper lobectomy

The postoperative CT imaging demonstrated a significant hypodense region within the left cerebral hemisphere (Figure 3a). Subsequently, a contrast-enhanced CT scan conducted eight days after the LUL revealed a 20-mm thrombus in the PVS (Figure 4a). Consequently, we commenced oral edoxaban 60 mg/day administration. The subsequent MRI delineated an extensive MCA territory infarction accompanied by intracerebral hemorrhage (Figure 3b, 3c). Given the effective medical management of the hemorrhage and swelling, surgical intervention for decompression was deemed unnecessary. Follow-up imaging indicated no thrombotic recurrence. A contrast-enhanced chest CT scan 18 days post-VATS LUL displayed a reduction in thrombus dimension (Figure 4b). The patient was transitioned to a convalescent facility on day 190 post-embolization, with a modified Rankin Scale score of 4.

## Discussion

This report examines a rare case of cerebral embolism originating from the PVS after LUL, leading to obstruction in the CCA. The focus is on two critical aspects: managing cerebral embolism associated with the PVS and applying DSRT for a challenging case of carotid bifurcation occlusion.

Regarding the management of cerebral embolism associated with the PVS, a pivotal aspect involves the cautious use of anticoagulants. Although these medications are traditionally employed to mitigate the development of thrombus, their administration following lobectomy requires careful consideration due to the potential risk of significant hemorrhage, a complication that can severely impact patient outcomes. There is no established consensus regarding the management of patients with PVS thrombosis, including the necessity of intervention [9]. The routine administration of anticoagulants following surgical procedures is also not standardized. Consequently, in the current scenario, early detection and treatment of thrombosis caused by the PVS are paramount, including early MT as a beneficial treatment option.

Collaboration between medical staff involved in stroke care and thoracic surgeons is also essential, particularly in the management of patients undergoing LUL, which is associated with PVS thrombosis in 17.9% of cases [3]. Notably, the risk of stroke is significantly higher at 5.17% in patients undergoing LUL compared to other lung resection methods [1]. This cooperative effort is key to accelerating treatment and enhancing outcomes for patients. It encompasses the preoperative sharing of patient information and the formulation of strategies to address any emerging neurological symptoms following the procedure. By adopting this approach, there is a potential that better treatment outcomes could have been achieved in our case as well.

For the prevention of recurrence of PVS thrombosis, treatment generally involves anticoagulation using heparin and warfarin. However, in our cases, we were able to achieve thrombus reduction and prevent recurrence for six months using direct oral anticoagulants (DOACs). Reports by Tsuji et al. [10] and Morinaga et al. [6] have indicated success in preventing the recurrence of PVS thrombosis using DOACs, underscoring the need for further research in this area.

For challenging thrombosis in the cervical common or internal carotid artery, particularly characterized by thrombi formed in the PVS, we utilized the DSRT with gradual simultaneous retraction using two Solitaire 6 × 40 mm SRs (Medtronic) and REACT 71 (Medtronic). Recent literature has highlighted the efficacy of the double SR technique for particularly difficult cases of MT at the top of the ICA or M1 bifurcation [7,8,11-15]. Sasaki et al. [7] and Imahori et al. [8] have described the double SR technique as a deliberate alteration in the trajectory of the devices, tailored to the geometry of the vessel, thereby enhancing the capacity for clot engagement. This approach facilitates the interaction between the device and the clot within the vessel and is posited as an effective rescue strategy for intracranial ICA occlusions [14].

Although the utility of the double SR technique has been documented for intricate intracranial scenarios, its application in carotid artery stenosis, particularly within the cervical ICA or CCA, remains undocumented. Moreover, no accounts have detailed its use in occlusions caused by PVS thrombosis. In instances of embolic events at the carotid bifurcation or within the extracranial segment of the ICA, the emergent clots are often of greater size, and the diameter of the vessel is larger than that found in intracranial arteries. In these instances, the double SR technique is advantageous, which can accommodate clots of various sizes and adapt to different arterial diameters, ensuring secure retrieval of the occlusive material. It is known that LUL, compared to other lobectomies, results in a longer stump [16], which could potentially contribute to the formation of the challenging, giant, and hard thrombi we have experienced. This unique aspect of LUL may play a role in the development of such difficult-to-treat thromboses, indicating the need for special consideration in these patients.

While the DSRT offers significant benefits in managing complex thrombectomy cases, it is essential to be aware of potential complications that may arise. In our case, the occlusion at the ICA top that occurred after DSRT is likely due to distal embolization during the DSRT procedure, potentially hindering the improvement of the patient's prognosis. This possibility is supported by the preoperative MRA, which showed that the left MCA area was visualized through cross-flow via the Acom. Although there have been no reports focusing on the possibility of distal embolization related to DSRT, our case highlights that DSRT may induce the collapse of thrombus. This might be particularly true for hard and large thrombi like in our case or those occurring after LUL following PVS thrombosis.

We must also consider the inherent risks associated with DSRT, including potential vascular damage, which might be elevated compared to conventional single SR use. Nevertheless, significant complications associated with the DSRT have not been reported in extant case studies. To mitigate procedural complications such as dissection, endothelial wall damage, and small artery avulsion, meticulous execution of thrombectomy procedures is imperative. Moreover, inherent risks such as stent fracturing and increased costs, when compared with standard techniques, necessitate a judicious assessment to determine the appropriateness of the DSRT for individual patient cases. The procedural risks may be reduced by employing gradual and simultaneous retraction maneuvers within the BGC [8]. The DSRT holds the potential for treating challenging thrombectomy cases; however, its effectiveness and safety require further validation through research and careful clinical application. To date, its use in cases of carotid bifurcation embolism due to PVS thrombosis after LUL remains undocumented, which indicates a need for more extensive research to establish its clinical utility in such scenarios.

## Conclusions

This report emphasizes the importance of teamwork between thoracic surgeons and medical staff involved in stroke care when dealing with the complex issue resulting from PVS embolization post-LUL. While the DSRT may offer a potential solution for AIS caused by a large thrombus, following embolization from a PVS after LUL, further accumulation of cases and investigations are needed to fully understand the risks of distal embolization, potential complications, and cost-effectiveness.
